# DNA, Cell Wall and General Oxidative Damage Underlie the Tellurite/Cefotaxime Synergistic Effect in *Escherichia coli*

**DOI:** 10.1371/journal.pone.0079499

**Published:** 2013-11-18

**Authors:** Roberto C. Molina-Quiroz, David E. Loyola, Claudia M. Muñoz-Villagrán, Raquel Quatrini, Claudio C. Vásquez, José M. Pérez-Donoso

**Affiliations:** 1 Laboratorio de Microbiología Molecular, Departamento de Biología, Facultad de Química y Biología, Universidad de Santiago de Chile, Santiago, Chile; 2 Laboratorio de Ecofisiología Microbiana, Fundación Ciencia y Vida, Santiago, Chile; 3 Microbiology and Bionanotechnology Research Group, Laboratorio de Bioquímica, Departamento de Bioquímica y Biología Molecular, Facultad de Ciencias Químicas y Farmacéuticas, Universidad de Chile, Santiago, Chile; 4 Universidad Andres Bello, Facultad de Ciencias Biológicas, Center for Bioinformatics and Integrative Biology (CBIB), Bionanotechnology and Microbiology Lab, Santiago, Chile; Queen’s University Belfast, United Kingdom

## Abstract

The constant emergence of antibiotic multi-resistant pathogens is a concern worldwide. An alternative for bacterial treatment using nM concentrations of tellurite was recently proposed to boost antibiotic-toxicity and a synergistic effect of tellurite/cefotaxime (CTX) was described. In this work, the molecular mechanism underlying this phenomenon is proposed. Global changes of the transcriptional profile of *Escherichia coli* exposed to tellurite/CTX were determined by DNA microarrays. Induction of a number of stress regulators (as SoxS), genes related to oxidative damage and membrane transporters was observed. Accordingly, increased tellurite adsorption/uptake and oxidative injuries to proteins and DNA were determined in cells exposed to the mixture of toxicants, suggesting that the tellurite-mediated CTX-potentiating effect is dependent, at least in part, on oxidative stress. Thus, the synergistic tellurite-mediated CTX-potentiating effect depends on increased tellurite uptake/adsorption which results in damage to proteins, DNA and probably other macromolecules. Our findings represent a contribution to the current knowledge of bacterial physiology under antibiotic stress and can be of great interest in the development of new antibiotic-potentiating strategies.

## Introduction

Multi-antibiotic resistance in bacteria has become a public health concern and also an important veterinary problem worldwide. Scientific and pharmaceutical efforts have been devoted to look for new antibiotics and to synthesize or modify existing ones. In spite of these efforts only one novel antibiotic has been introduced in the market during the last 50 years [1]. In addition, even if new antibiotics become available the emergence of resistant strains is only a matter of time.

A few alternative strategies to potentiate the antibacterial effect of known antibiotics have been proposed. Increasing antibiotic toxicity in a synergistic manner combining those with chemical compounds or metals having different cellular targets are among the most promising ones. This strategy significantly reduces the probability of emergence of strains that are resistant simultaneously to both antibacterials. This is the case of tomatidine, a natural compound that acts synergistically with aminoglycosides against multiresistant strains of *Staphylococcus aureus* [2]. In the same line, while bismuth thiols have been used to improve tobramycin effects [3], the combined administration of the complex desferrioxamine- gallium and gentamicin increased the toxicity against *Pseudomonas aeruginosa* [4].

In spite of recent contributions in this field, much remains to be explored regarding the molecular mechanisms underlying antibiotic toxicity and synergistic effects. The picture supporting a relevant role for oxidative stress in general toxicity of bactericidal antibiotics [5] has been recently challenged [6], [7].

To the best of our knowledge, studies regarding the elucidation of molecular mechanisms involved in antibiotic potentiation have not been published to date. Recently, our group reported the successful use of non-lethal concentrations of the tellurium oxyanion, tellurite (TeO_3_^2−^), to potentiate antibiotic toxicity. A tellurite dose-dependent effect on the toxicity of several antibiotics was observed in both clinical and laboratory strains. Particularly interesting was the synergistic effect observed with TeO_3_^2−^ and the widely used third-generation cephalosporin, cefotaxime (CTX) [8].

At very low (nM) concentrations, tellurite acts as a strong toxicant for bacteria exerting its toxic effects essentially through the generation of oxidative stress that in turn oxidizes proteins, membranes, depletes the reduced-thiol pool and affects metabolic enzymes [9].

Although tellurite has been used as selective agent for decades in routine microbiological culture media, information about tellurite damage/toxicity in cells of superior eukaryotes is scarce. Only a couple of representative reports are available including murine hepatocarcinoma transplantable liver tumor (TLT) cells [10] and whole animal assays (rats) [11]. Thus, the combined approach tellurite/antibiotic seems particularly attractive for antibiotic-potentiating strategies.

In this work we investigated the molecular mechanism(s) underlying the potentiating effect of TeO_3_^2^ on CTX in *E. coli*. Global transcriptional profiling using microarrays and cellular damage assessment indicated that in the presence of CTX increased uptake of tellurite takes place with the concomitant oxidative damage of proteins and DNA. These findings may represent the basis for new strategies to potentiate the antibacterial effect of current antibiotics as well as a source of putative targets for the development of new ones.

## Results

### Global Expression Experiments

Global changes in the transcriptional profile of *E. coli* exposed to different conditions were assessed using DNA microarrays as described in Methods. Induced/repressed genes in each particular experimental condition were 19/24 (tellurite), 32/84 (CTX) and 29/33 (tellurite/CTX). The observed fold-change for induced and repressed genes in the presence of the toxicants was: 4.7/2.1 (tellurite, induced) and −3.5/−2.0 (tellurite, repressed); 4.5/2.0 (CTX, induced) and −3.9/−2.0 (CTX, repressed); 7.8/2.0 (tellurite/CTX, induced) and −5.2/−2.0 (tellurite/CTX, repressed) (Table S2).

In order to identify the cell pathways that are affected in response to tellurite, CTX and/or tellurite/CTX exposure, induced and repressed genes in each condition were grouped using Gene Ontology (GO) terms. In all cases the three gene categories most represented in the subset of differentially expressed genes were stress responses, transport and biosynthetic processes (Fig. S1 A, B, C).

Induced genes in tellurite-exposed cells belonged mainly to stress responses, biosynthetic and metabolic processes, transcription and RNA catabolism and protein metabolism categories. Most highly expressed genes in this condition included well recognized oxidative stress markers, confirming previous results. Even though the concentration of tellurite used in this study was ten-fold lower than that in previous experiments [12], exposure to the toxicant still resulted in *soxS* gene induction (GeneID: 948567, 4.7 fold-change), along with that of others such as *marR* and *dnaK* (GeneIDs:945825 and 944750, each with almost 3 fold-change), related to metal response and oxidative stress, respectively [13], [14] (Table S2 A). Induction of genes related with protein folding and RNA degradation was also observed (Fig. S1 A, Table S2 A) suggesting that tellurite exposure results in oxidative damage to protein and RNA components.

Upon CTX-exposure most induced genes were related to transport, including *yhdA*, *yaaH*, *ydgK*, *yjgP*, *nikC*, *ygeD*, *secG*, *smpA* and *ynfM* that encode different conserved and predicted inner membrane proteins, nickel transporter subunit and other transporters (Fig. S1 B, Table S2 B). In addition, induction of genes involved in DNA repair such as *uvrA* and *pol* [both participating with an oxidative damage-dependent DNA repair system [15] was also observed (Table S2 B). Interestingly, *intZ* (CPZ-55 prophage predicted integrase) and *ycfK* (e14 prophage predicted protein) were also induced, suggesting the activation or induction of a putative phage excision mechanism, as suggested previously [16]. The results presented above strongly suggest that CTX produces oxidative damage to DNA. This is most probably mediated by hydroxyl radicals, in a similar manner to that described previously for bactericidal antibiotics [5].

As with tellurite, simultaneous exposure of *E. coli* to tellurite/CTX results in the induction of genes (*soxS*, *marR* and *dnaK)* that are associated with the response to oxidative stress [12], [13], [14], but also others related with transport and biosynthetic processes (Fig. S1 C). Induction of genes related to protein folding and [Fe-S] clusters repair also supports the idea that part of tellurite/CTX-mediated damage results from oxidative stress. To facilitate data analysis of tellurite- and CTX-regulated genes, Venn diagrams were constructed (Fig. S4). In general, array data were validated by qRT-PCR as described in Methods (Fig. S3).

### Tellurite Concentration in Antibiotic-exposed *E. coli* Cultures

**Figure 1:**
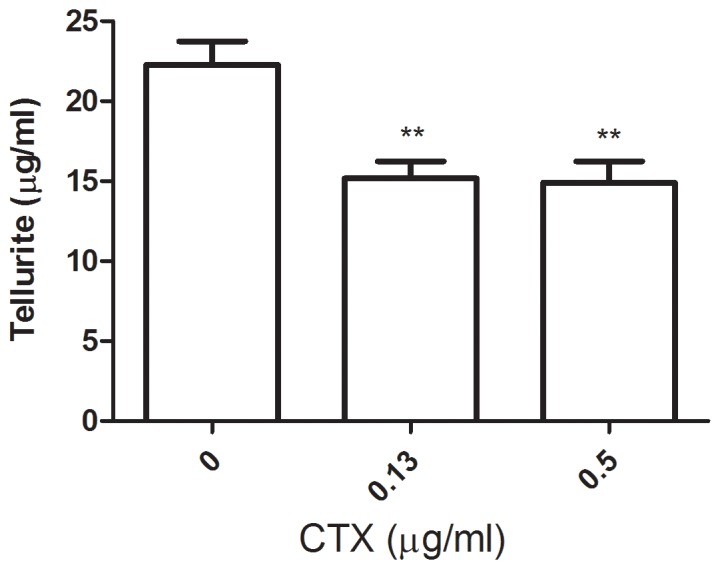
Extracellular tellurite concentration in culture supernatants. Tellurite concentration was determined in supernatants of *E. coli* cultures that had been exposed to tellurite (20 µg ml^−1^) in the absence (control) or presence of the indicated CTX concentrations. Values represent the average of 3 independent experiments. Statistical significance was determined using t-test. (**) p<0.01.

Since the canonical target of CTX is peptidoglycan, a possible explanation for the observed synergistic effect is that the destabilization of the cell wall caused by CTX may result in increased intracellular tellurite concentrations. Then, the simultaneous presence of both toxicants might cause exacerbated oxidative stress. To test this hypothesis, the cell wall integrity was assessed indirectly in *E. coli* exposed or not to the antibiotic through quantifying remaining tellurite in the culture medium as described in Methods. A ∼35% decrease of extracellular tellurite was determined in culture supernatants of cells exposed to lethal or non-lethal CTX concentrations (Fig. 1), providing evidence for an increase in tellurite entrance/adsorption to the cell in these conditions. In this context, preliminary experiments using FACE (Fluorescence Assisted Carbohydrate Electrophoresis [17]) with tellurite/CTX-exposed *E. coli* suggest enhanced activity of murein hydrolases, indicative of alterations in the regulation of normal peptidoglycan turnover (not shown).

### ROS Generation in Toxicant-exposed Cultures

Given that i) bactericidal antibiotics apparently generate hydroxyl radicals [5], ii) tellurite generates superoxide [12] and iii) the expression profile of tellurite/CTX cells suggest the occurrence of an exacerbated oxidative stress status, the generation of ROS in toxicant-exposed cells was evaluated. Preliminary experiments using the non-specific ROS probe H_2_DCFDA showed a slight increase of total ROS in cells exposed to sublethal CTX concentrations in the presence of tellurite (not shown).

Superoxide generation in cells exposed simultaneously to tellurite and different CTX concentrations was determined by flow cytometry using the superoxide-specific probe dihydroethidine. In turn, the OH^•^-specific probe hydroxyphenyl fluorescein (HPF) was used to assess OH^•^ generation in treated cells.

**Figure 2:**
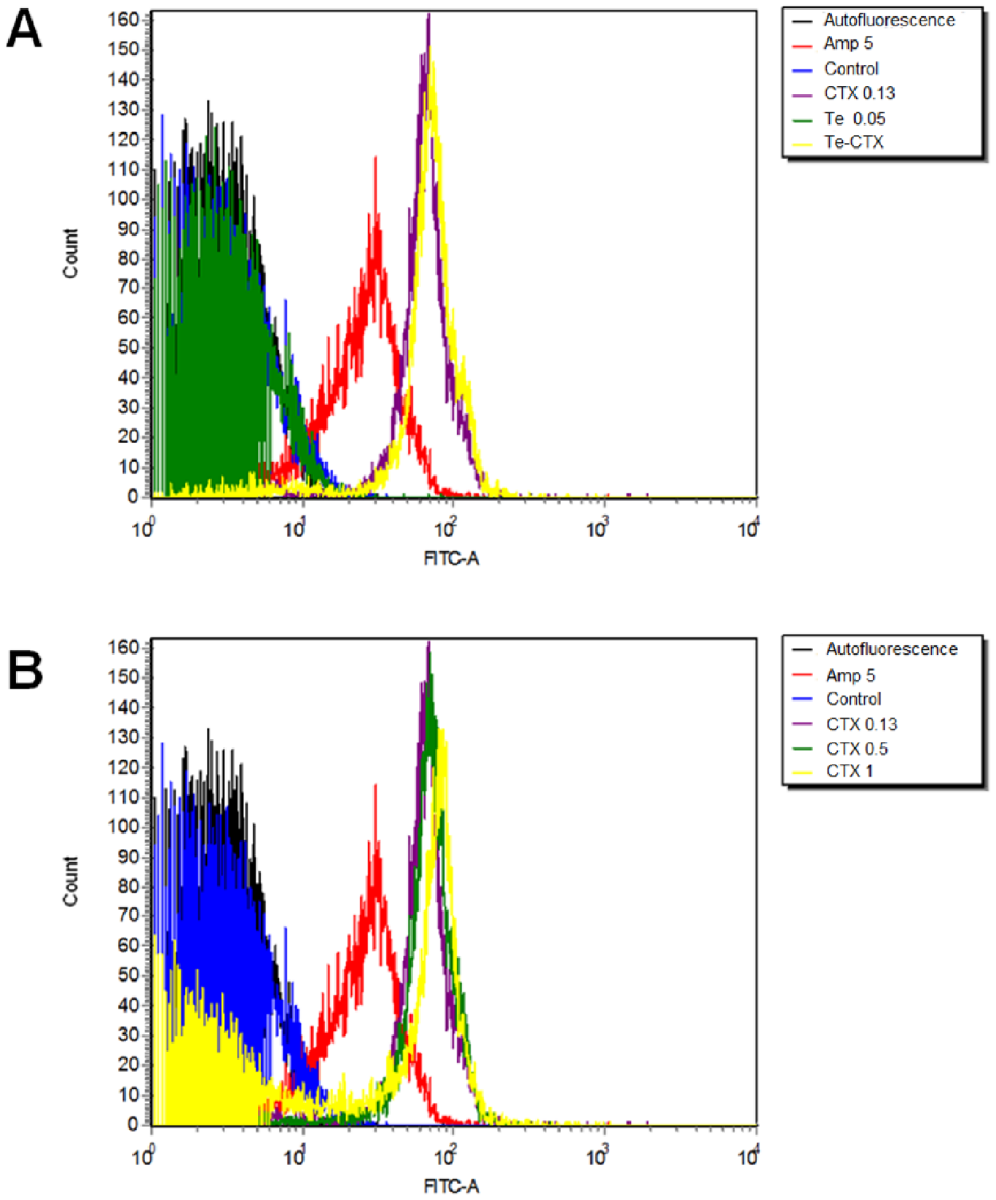
Hydroxyl radical generation in *E. coli* exposed to CTX or Te/CTX. *E. coli* was exposed for 3 h to tellurite/CTX (A) and to different antibiotic concentrations (B). As positive control, cells were exposed for 3 h to ampicillin. Units are expressed in µg ml^−1^. The data correspond to a representative result of 3 independent trials.

As shown in Fig. S2, in the presence of both toxicants superoxide levels are equivalent to those observed in cells exposed to tellurite alone (compare blue and green lines), demonstrating that superoxide anion is formed at short exposure times. Also, a significant increase in OH^•^ levels was observed in *E. coli* after 3 h exposure to tellurite/CTX (Fig. 2) mimicking the effects of exposure to CTX alone. These results demonstrate that tellurite contribution to the tellurite/CTX combined effect does not proceed through OH^•^ generation, but rather through superoxide. The observed effects are independent of the CTX concentration (Fig. 2 B). Conversely, CTX contribution to the tellurite/CTX effect seems to proceed through OH^•^ generation in a dose dependent manner (Fig. 2 B).

### ROS-induced Damage to Cell Macromolecules

**Figure 3:**
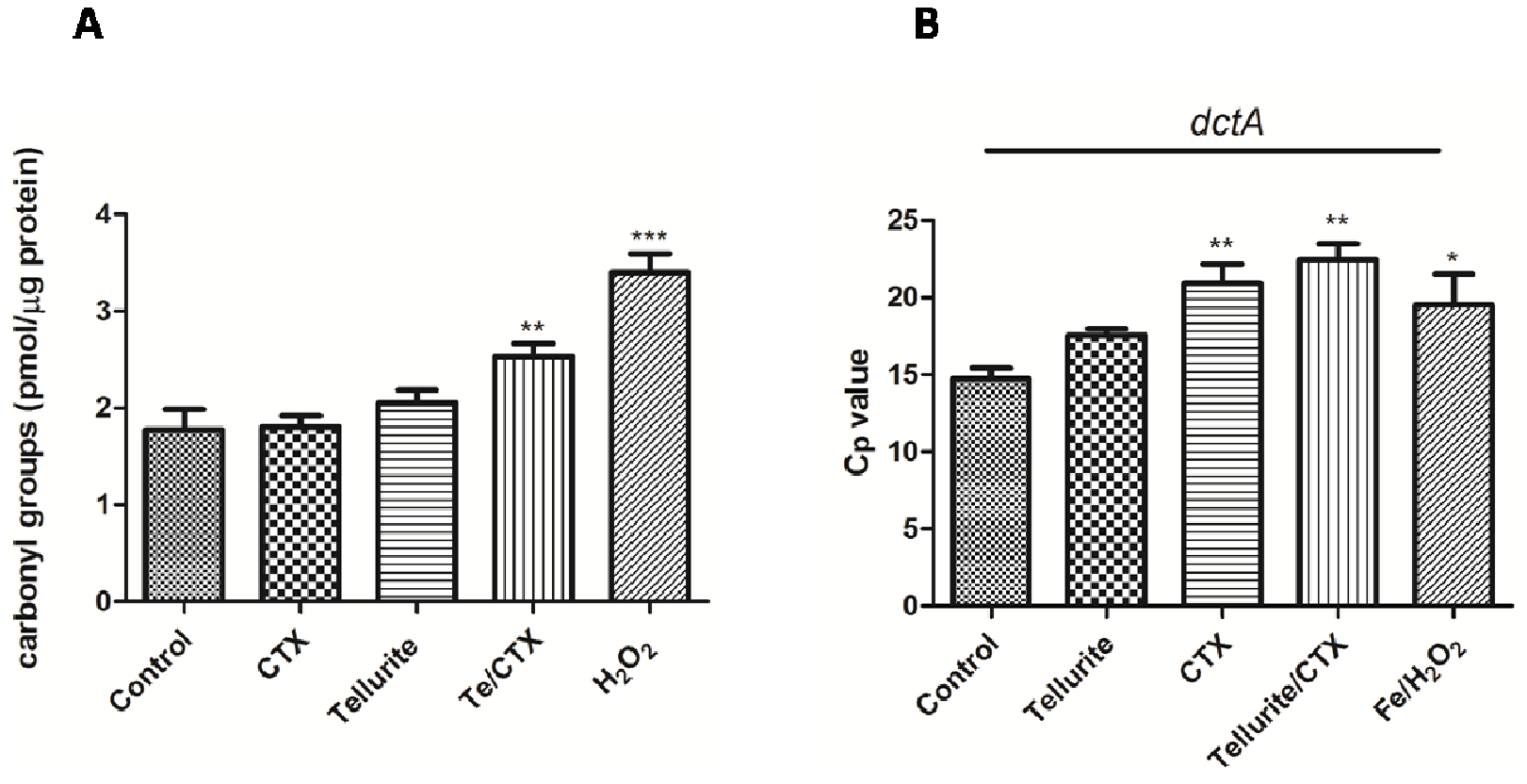
Tellurite/CTX-mediated damage to macromolecules. **A,** protein oxidation was assessed by determining the content of carbonyl groups after 15 µg ml^−1^ tellurite, 0.5 µg ml^−1^ CTX or tellurite/CTX at the same concentrations. H_2_O_2_ (2.5 mM) was used as positive control of oxidation. **B,** DNA damage was determined by real time PCR using total DNA from cultures exposed during 3 h to 0.05 µg ml^−1^ tellurite, 0.13 µg ml^−1^ CTX or TeO_3_^2−/^CTX (at the same concentrations). FeSO_4_ and H_2_O_2_ (50 µM and 100 mM, respectively) were used as positive control of DNA damage (Fenton reaction).

Since ROS generated as consequence of tellurite/CTX exposure can randomly affect cell macromolecules, damage to both proteins and DNA was evaluated. Protein damage was assessed by carbonyl group formation. Enhanced levels of oxidized proteins were observed in cells exposed to tellurite/CTX but not in cells exposed to either toxicant alone (Fig. 3A). In turn, DNA damage was assessed by real time PCR. In order to cover a representative portion of the bacterial chromosome, 5 randomly-selected genes were amplified. A representative experiment using the *dctA* gene is shown in Fig. 3B. Higher C_p_ values were observed in CTX- and tellurite/CTX-treated cells as compared to controls, indicating a direct OH^•^-mediated injury to the DNA molecule (Fig. 3B).

## Discussion

The continuous emergence of antibiotic-resistant bacteria which affects not only public health but also veterinary and food industries has become an important scientific concern during the last years. Aiming to explore new alternative antibacterial treatments, combination of different antibiotics [18] or the administration of defined molecules acting as adjuvants have been proposed [19], [20]. In this context, the antibiotic-potentiating effect of potassium tellurite at concentrations that are not toxic to eukaryotic cells has been reported [8]. To elucidate the molecular mechanism(s) involved in this phenomenon, global transcriptomic changes in *E. coli* exposed to tellurite, CTX or TeO_3_^2−/^CTX were evaluated.

### Tellurite-induced Changes in Gene Expression

One of the most obvious results of gene expression profile analysis upon toxicant treatment was the induction of stress-responsive genes, many of which play a pivotal role in the oxidative stress response. For instance, induction of *soxS* and *fumA* was observed (Table S1), which agrees with previous results from our laboratory [21]. Since FumA, the gene product of the *fumA* gene, is an oxidative stress-sensitive relevant TCA cycle enzyme [21], [22], [23], its induction suggests that compensatory effects to restore ROS-damaged enzymes are in place. In addition, induction of *dnaK*, encoding the DnaK chaperone that participates in different stress response pathways including heat shock [14] and antioxidant response against H_2_O_2_ [24], *yfhQ*, which has been directly related to DNA repair under ROS-mediated stress in *Bacillus subtilis* [25] and *marR*, a transcriptional regulator that has been associated with oxidative stress and response to antibiotics was also observed [13]. The induction of these -and- other genes may be related to the tellurite-induced superoxide stress.

On the other hand, the analysis of expression data showed that most highly expressed and repressed genes were related to diverse biosynthetic processes (Fig. S1). One example is the repression of *dcuS*, *glpB* and *narG* genes, all related to anaerobiosis in *E. coli*, which suggests a preference for aerobic metabolism in the presence of tellurite. In turn, upregulation of *acnA*, *fumC* and *zwf*, encoding aconitase A, fumarase C and glucose 6 phosphate dehydrogenase, respectively (a consequence of *soxS* induction), also supports a preference for aerobic metabolism in tellurite-exposed cultures.

Repression of *rseB*, encoding a negative regulator of *rpoE* (σE), was also observed. Since induction of *rpoE* is consequence of the erroneous folding of periplasmic proteins [26], the observed *rseB* repression suggests that correct folding of these proteins may be accomplished by tightly regulating *rpoE* expression, as previously reported [27].

Changes in the global transcription profile of *E. coli* exposed to 0.5 µg/ml tellurite were also assessed by DNA microarray analysis. Surprisingly induction of *soxS*, *gmk*, *marR* and *cspA* was still observed at this tellurite concentration (10-fold higher than those used in most experiments shown in this work). The *E. coli* transcriptional response to tellurite-mediated stress seems to be dependent on the oxyanion concentration, as a dose-dependence of the log-fold change value was observed for the referred genes (Table S3).

Despite cells being exposed to a sublethal tellurite concentration (0.05 µg/ml), resulting transcriptional changes suggest that pleiotropic damage occurs and affects mainly the central metabolism, most probably via the generation of oxidative stress.

Some work on transcriptional profiling in tellurite-exposed *E. coli* has been recently published [28]. Comparable changes to those reported here were observed by these authors regarding carbohydrate and amino acid metabolism. However, lack of other similarities is most probably due to the different experimental conditions used. For instance, *E. coli* EC3 instead the BW25113 strain was their model organism and tellurite concentrations were 100-fold higher [28] than those used here.

### CTX-induced Changes in Gene Expression

Global gene expression analysis in *E. coli* suggest that CTX exposure results in the induction of genes involved in the stringent response, oxidative stress-mediated DNA damage repair and in *de novo* synthesis of membrane proteins. CTX exposure resulted in the induction of *spoT*, *ygjM* and *guaC* genes which are related to the stringent response. This is a condition of amino acid starvation, also triggered by other kinds of stresses that result in the inhibition of several metabolic processes, thus allowing bacterial survival. It is characterized by a rapid accumulation of guanosine 3′–5′-bispyrophosphate (ppGpp), a nucleotide that regulates positively or negatively the expression of different gene sets [29], [30], [31].

Expression of several genes involved in ppGpp accumulation, σ^E^-mediated envelope stress, heat shock, osmotic and oxidative damage have been shown to be induced upon exposure to cell wall-acting antibiotics in *Streptomyces coelicolor* [32]. These results are in agreement with our findings in differential expression (Table S2; Fig. S1B). Moreover, induction of *uvrA* (nuclease A) and *polA* (DNA polymerase I) genes, involved in the nucleotide excision DNA repair mechanism (UvrABC) [33], was observed in CTX-treated cells. This result is supported by the observation of Kohanski and coworkers regarding hydroxyl radical generation by bactericidal antibiotics [5].

Notably, most induced genes were grouped in the transport functional category, suggesting that the antibiotic-induced damage to the cell wall causes not only its destabilization but could also result in membrane protein misfolding. A similar envelope effect was reported for thioridazine-treated *Mycobacterium tuberculosis*, where induction of membrane protein genes occurred simultaneously with cell wall damage [34].

### Changes of Gene Expression in the Presence of Tellurite/CTX

As with tellurite alone, *soxS*, *marR*, *dnaK* and *yfhQ* genes (all related to the oxidative stress response) were also found to be induced in tellurite/CTX-exposed cells. This result was not unexpected since tellurite exposure results in the generation of an oxidative stress status in *E. coli* [9], [12]. In addition, induction of *clpB* (encoding the caseinolytic peptidase B) and *dnaK* (encoding the DnaK chaperone) also occurred in the presence of both toxicants, again suggesting that a certain degree of protein misfolding is taking place in this condition. DnaK, along with IbpA and IbpB proteins, forms a triad that is responsible for solubilizing thermally-denatured protein aggregates *in vivo* and *in vitro* [35]. On the other hand, the induction of the *yaeL* gene, encoding the extracytoplasmic stress-responsive σ^E^-activating proteinase [36], suggests that these cells are also facing periplasmic stress. This would be a consequence of CTX-mediated damage to the cell wall and/or oxidative damage to membrane and membrane proteins. Functional assignment analysis of genes expressed in tellurite/CTX treated cells clearly suggests that pleiotropic damage provoked by exacerbated oxidative stress is occurring in these conditions.

As shown in Tables S1–S4 the number of tellurite-, CTX- and tellurite/CTX-regulated genes is low (especially evident in Fig. S4). Our interpretation is that this observation is due most probably to the strict cut criteria used to interpret the microarray analysis data.

### Identification of Tellurite/CTX Cell Targets

As in the case of other cephalosporin antibiotics, CTX-mediated damage to the cell wall is well known and in this scene, increased uptake/adsorption of tellurite may be expected. Indirect evaluation of tellurite uptake in tellurite/CTX-exposed cells -using a previously reported strategy [37]- proved this to be the case although this effect did not depend on CTX concentration (Fig. 1).

Higher levels of intracellular tellurite in the presence of CTX are thus likely to exacerbate the production of ROS and induced ROS-mediated damage to macromolecules. ROS were produced in both tellurite- and tellurite/CTX-treated cells as assessed through the use of the nonspecific H_2_DCFDA probe. Based on these results, the identity of the specific oxygen radical involved in the tellurite/CTX synergy phenomenon was determined. Using DHE, superoxide was detected even at tellurite concentrations that were 10-fold lower than those used previously [12]. Interestingly, superoxide is not generated in CTX-exposed cells, suggesting that only tellurite is responsible for early ROS generation in tellurite/CTX-exposed *E. coli*. On the other hand, hydroxyl radical production was investigated in tellurite/CTX-treated cultures. After 3 h of exposure to the toxicants, OH^•^ levels were similar in CTX- and tellurite/CTX-challenged cells (Fig. 2A). Also, they did not change significantly when cells were exposed to different antibiotic concentrations, although a slight displacement of the fluorescence peak could be appreciated (Fig. 2B). Thus, tellurite and CTX seemed to be the main responsible for superoxide and hydroxyl radical, respectively. Surprisingly, fluorescence in CTX- or tellurite/CTX-exposed cells was higher than that observed with ampicillin-treated cells (positive control). This should not be a consequence of the total number of antibiotic molecules since ampicillin concentration was 50-fold higher than that of CTX. Although these 2 bactericidal antibiotics share the same cell target, these results suggest that both have different OH^•^-generating mechanisms.

To evaluate superoxide- and OH^•^-induced cell injury, direct damage to proteins and DNA was then assessed. Increased levels of protein carbonylation were observed exclusively in tellurite/CTX-exposed cells (Fig. 3A), showing higher protein oxidation than that observed with tellurite or CTX alone. In turn, higher qPCR crossing points (Cp values) were obtained in CTX- and tellurite/CTX-exposed cultures, indicating that oxidative injury to the cell’s DNA occurred (Fig. 3B).

Finally and although the OH·-detecting methodology was challenged recently [38], our trancriptomics and DNA damage data support the notion that hydroxyl radicals are being produced upon tellurite/CTX exposure.

### Concluding Remarks

**Figure 4:**
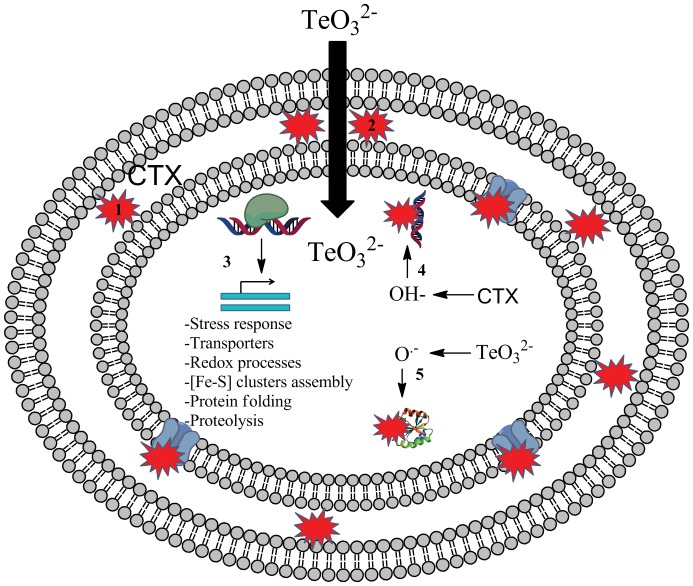
Tellurite/CTX damage in *E. coli*. Peptidoglycan integrity (stability) is affected by CTX (1), favoring tellurite entry (2). Tellurite/CTX administration generates global transcriptional changes on different stress response pathways, transport, [Fe-S] clusters assembly, protein folding and different oxidative stress regulators (3). Finally, CTX and tellurite generate hydroxyl radical and superoxide respectively, damaging DNA, proteins and most probably other macromolecules.

Results from this work show that the tellurite/CTX-challenge alters the global gene expression profile and produces cellular damage. Increased levels of intracellular superoxide and OH^•^ produced by tellurite and CTX, respectively, generate direct damage to DNA (and probably RNA) and to the protein pool, which may include a number of membrane transporters. This would favor further entry of tellurite and CTX, and sustain oxidative stress in time. All these effects would explain, at least in part, the synergistic CTX/tellurite phenomenon in *E. coli*. Intracellular ROS also trigger the expression of oxidative and envelope stress-related genes which result in the synthesis of DNA repair enzymes and membrane proteins (Fig. 4). Experiments to shed further light to the synergistic action of CTX and tellurite in *E. coli* are in progress in our laboratory.

## Materials and Methods

### Bacterial Strains and Culture Conditions

Unless otherwise stated, *E. coli* BW25113 (*rrnB3* Δ*lacZ4787 hsdR514* Δ*(araBAD)567* Δ*(rhaBAD) 568 rph-1*) was routinely grown in Luria Bertani broth at 37°C with shaking in the presence or absence (control) of 0.05 µg/ml tellurite (1/20 MIC) and/or 0.13 µg/ml CTX, as described [8]. This strain exhibits the same CTX MIC as pathogenic, ampicillin-resistant clinical *E. coli* isolates [39]. When required, ampicillin (50 µg/ml) was added to the medium. Tellurite concentration was assessed as described [37] .

### DNA Microarray Experiments

Total RNA was extracted from *E. coli* cultures exposed for 15 min to tellurite, CTX or tellurite/CTX using RNeasy Mini kit (Qiagen), as recommended by the manufacturer. RNA concentration and purity was determined using a Nanodrop 2000c spectrophotometer (Thermo).

Labeled cDNA probes were generated by reverse transcription using 20 µg of total RNA, SuperScript II (Invitrogen) and Alexa 555 and 647 dyes (Invitrogen) by conventional methodologies. Dye incorporation into cDNA was measured spectrophotometrically and generated probes were used to hybridize DNA microarrays slides purchased to Microarrays Inc as described earlier [40], with minor modifications. Slides contained 9,308 oligonucleotides representing four *E. coli* varieties and three plasmids associated to this bacterium. In particular, printed oligonucleotides corresponded to 4,269, 5,306, 5,251 and 5,366 open reading frames from *E. coli* K12, 0157:H7 (EDL933), 0157:H7 (Sakai) and CFT073 strains, respectively. In addition, 3, 10 and 97 genes from plasmids OSAK1, pO157_Sakai and pO157_EDL933, respectively, were also present in the slides. These were scanned in a ScanArray G_X_ (Perkin Elmer) scanner and image analysis was conducted using GenePix Pro v6.0 software. To discount the background signal we utilized the Bioconductor software and values were normalized using the LOESS method. T-test was used to identify those genes whose expression changed significantly upon treatment. Finally, 3 criteria to identify genes with differential expression (M value, A value and p value from t-test) were used. Only those genes that exhibited values of M ≥1 (induction), M ≤ −1 (repression); A ≥8 and p≤0.05 were considered [41]. Microarray data were deposited as GSE39696 series in GEO (Gene Expression Omnibus) at the NCBI’s database.

Microarray data was validated by qRT-PCR using *fadD* as the housekeeping gene (C_p_ values did not change in all conditions tested). Specific primers used are indicated in Table S1. A 300 bp DNA fragment of each gene was amplified using Kapa Sybr® Fast kit (KapaBiosistems) in a Rotorgene Q (Qiagen) thermocycler. Data generated was analyzed using the relative expression software tool (REST) [42].

### ROS Detection

*E. coli* cultures were grown to OD_600_ ∼ 0.4 and incubated with TeO_3_^2−^, CTX or both compounds simultaneously. Total ROS, superoxide and hydroxyl radical generation was assessed using 2′,7′-dichlorodihydrofluorescein diacetate (H_2_DCFDA), dihydroethidium (DHE) and hydroxyphenyl fluorescein (HPF), respectively, as described previously [5], [43]. Fluorescence of cultures exposed to antibacterial agents was determined by flow cytometry in a Becton Dickinson FacsCanto II citometer.

### Protein Carbonylation

Protein oxidation levels were assessed by determining spectrophotometrically the content of protein carbonyl groups as described previously [44].

### DNA Damage Determination

Total DNA was extracted from cells exposed to tellurite, CTX and TeO_3_^2−/^CTX during 3 h by conventional phenol:chlorophorm extraction methodologies [45] and 10 µg of the obtained material was used as template for real time PCR reactions using Kapa Syber® Fast kit (KapaBiosistems) as reported [46].

## Supporting Information

Figure S1 **Induced and repressed genes in the presence of tellurite (A), CTX (B) and tellurite/CTX (C) grouped according to Gene Ontology terms.**
(TIF)

Figure S2 **Determination of superoxide anion by flow cytometry using the specific probe dihydroetidine.** ***E. coli*** **cultures were exposed for 15 min to different CTX concentrations: sublethal (A), MIC (B) and lethal (C) in the presence or absence of tellurite (0.05 µg ml^−1^).** Units are expressed in µg ml^−1^. The figure corresponds to a representative result of at least 3 independent trials.
(TIF)

Figure S3 **Validation of microarray data. Gene induction in** ***E. coli*** **exposed to tellurite (A), CTX (B) or tellurite/CTX (C) as determined by qRT-PCR. Relative expression values were determined using the REST software** [**42**] **.** Statistical significance was assessed using the t-test. *p<0.05, **p<0.01, ***p<0.001.
(TIF)

Figure S4 **Differential gene expression in** ***E. coli*** **exposed to the indicated compounds.** Venn diagrams showing the number of induced (A) and repressed (B) genes that coincide between the indicated experimental conditions. CTX, cefotaxime.
(TIF)

Table S1 (DOCX)

Table S2 (DOCX)

Table S3 (DOCX)

Table S4 (DOCX)
