# Supplementary figures and images for: DNA, Cell Wall and General Oxidative Damage Underlie the Tellurite/Cefotaxime Synergistic Effect in Escherichia coli
Source: PLoS One. 2013 Nov 18;8(11):e79499. doi: 10.1371/journal.pone.0079499 (PMC3832599; doi:10.1371/journal.pone.0079499)

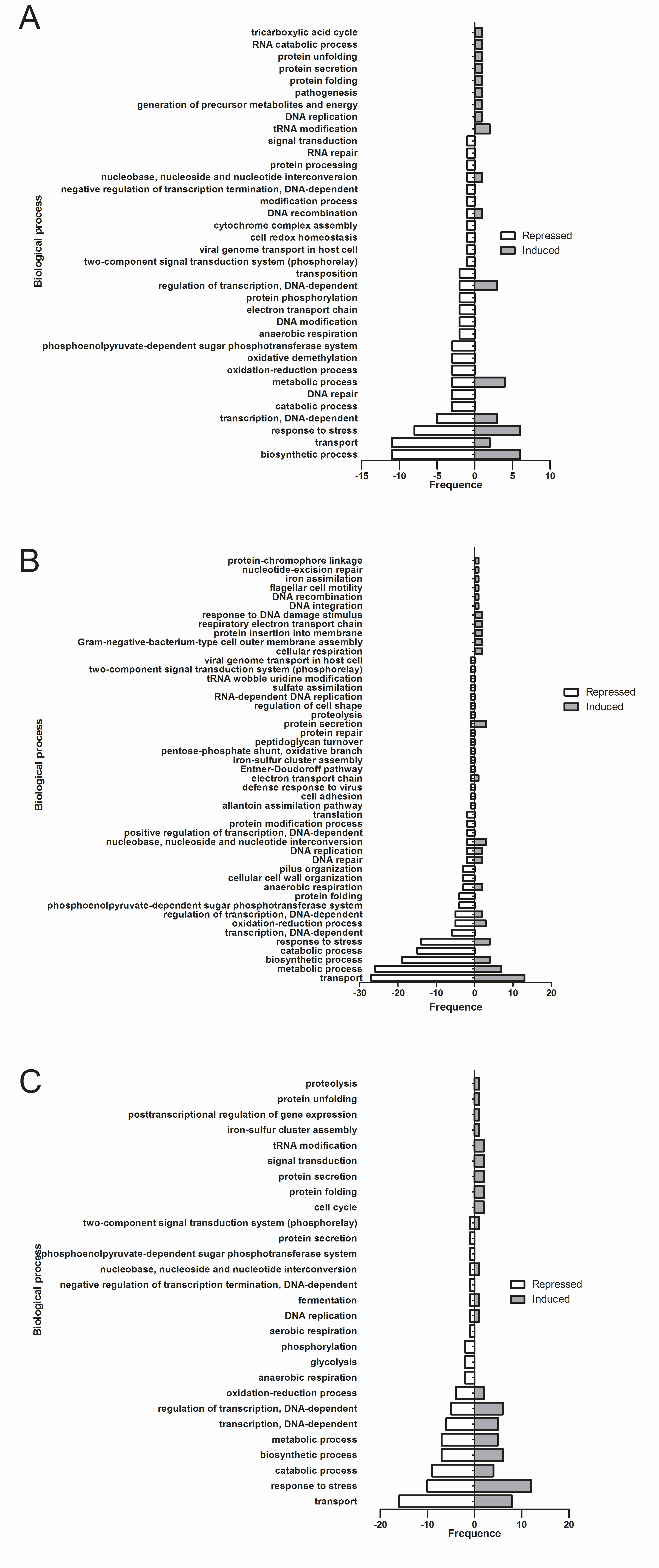

Supplement: Figure S1 — Induced and repressed genes in the presence of tellurite (A), CTX (B) and tellurite/CTX (C) grouped according to Gene Ontology terms. (TIF) [file pone.0079499.s001.tif]

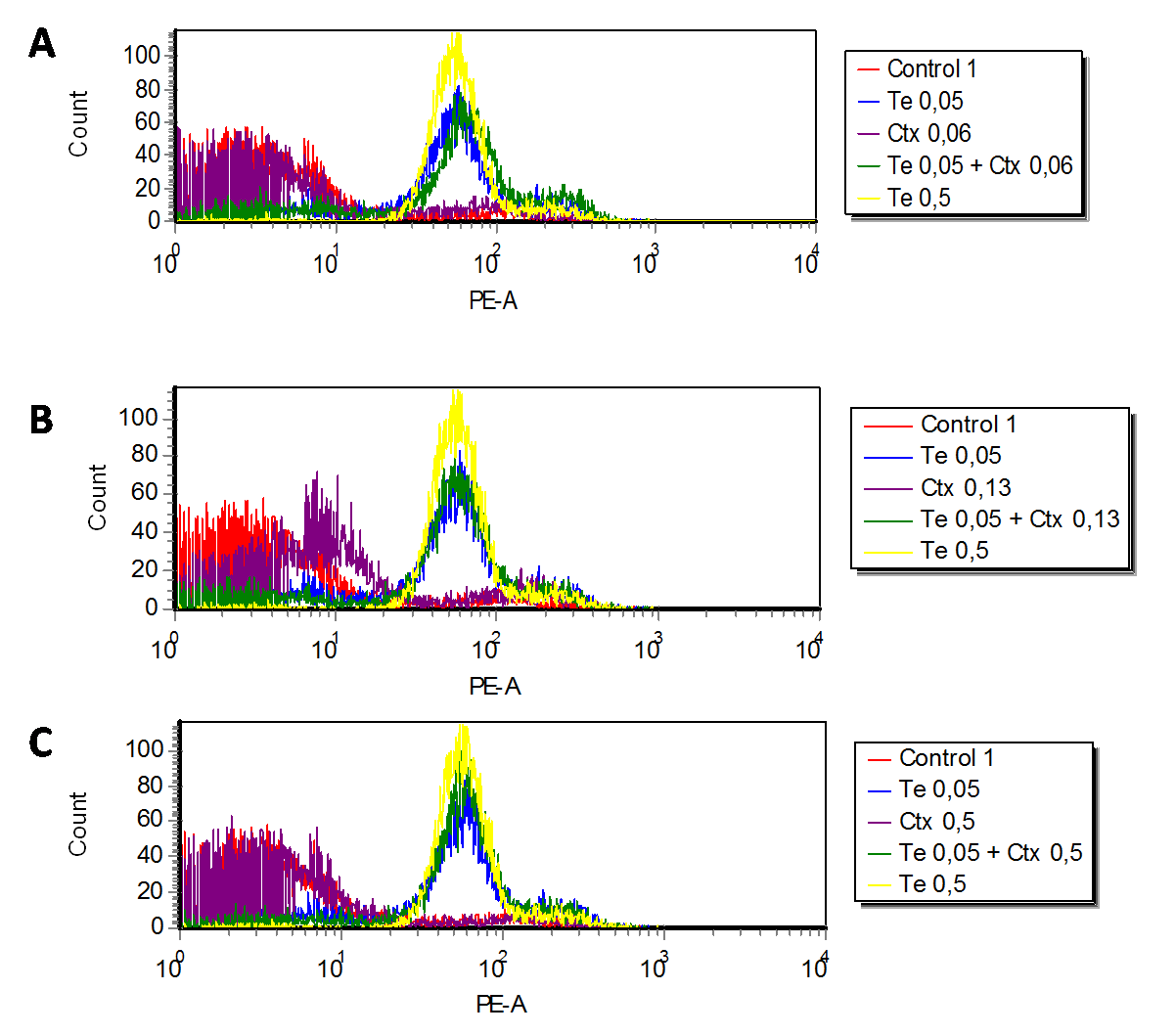

Supplement: Figure S2 — Determination of superoxide anion by flow cytometry using the specific probe dihydroetidine. E. coli cultures were exposed for 15 min to different CTX concentrations: sublethal (A), MIC (B) and lethal (C) in the presence or absence of tellurite (0.05 µg ml−1). Units are expressed in µg ml−1. The figure corresponds to a representative result of at least 3 independent trials. (TIF) [file pone.0079499.s002.tif]

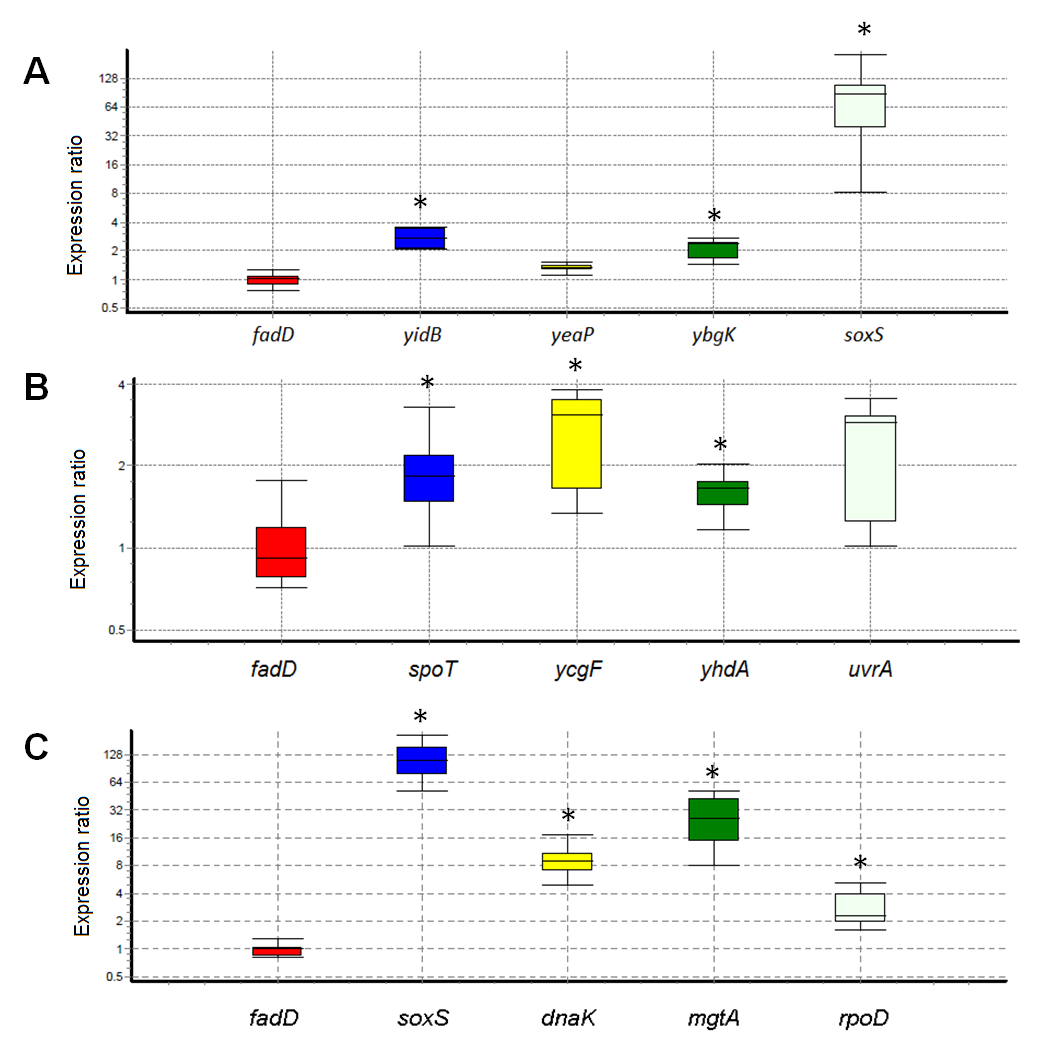

Supplement: Figure S3 — Validation of microarray data. Gene induction in E. coli exposed to tellurite (A), CTX (B) or tellurite/CTX (C) as determined by qRT-PCR. Relative expression values were determined using the REST software [42] . Statistical significance was assessed using the t-test. *p<0.05, **p<0.01, ***p<0.001. (TIF) [file pone.0079499.s003.tif]

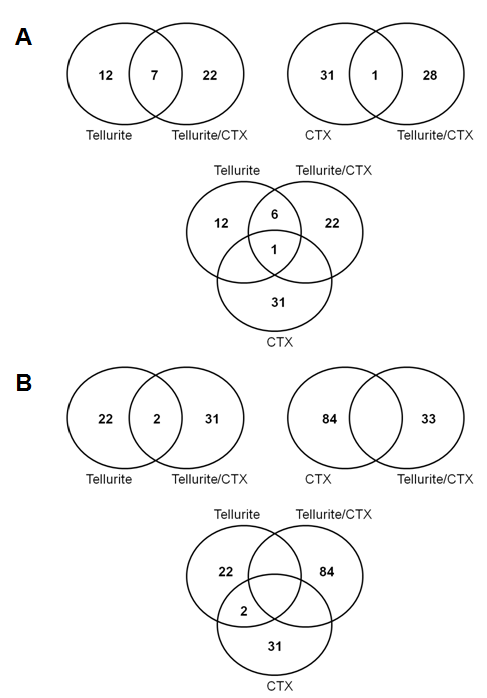

Supplement: Figure S4 — Differential gene expression in E. coli exposed to the indicated compounds. Venn diagrams showing the number of induced (A) and repressed (B) genes that coincide between the indicated experimental conditions. CTX, cefotaxime. (TIF) [file pone.0079499.s004.tif]
