# Supplementary material for: DNA, Cell Wall and General Oxidative Damage Underlie the Tellurite/Cefotaxime Synergistic Effect in Escherichia coli
Source: PLoS One. 2013 Nov 18;8(11):e79499. doi: 10.1371/journal.pone.0079499 (PMC3832599; doi:10.1371/journal.pone.0079499)
Supplement: Table S1 — (DOCX) [file pone.0079499.s005.docx]

**Table S1.** Primers used in this study

| Primer | Sequence (5´- 3´) |
| --- | --- |
| qF *yihQ* | AAGCTCGGCGAGATCCTTTT |
| qR *yihQ* | GTTGTCGCCAGGACGGTTA |
| qF *fadD* | TTCTCCAGTCTGCATCTTTCCG |
| qR *fadD* | ATCGGGACGCTGCCAGTAAC |
| qF *dctA* | GGTCATCTTCGGCATCATCA |
| qR *dctA* | TCTCCATCTTGTCGAGCATACG |
| qF *phnD* | TTGGCAATGGCGATCCTAAC |
| qR *phnD* | TGTCTTTGGTGGTTTCGGAA |
| qF *yhdA* | CAAGAATTGCTGGAAACCCG |
| qR *yhdA* | CGGCAGACGAATCATCATCA |
| qF *ycgF* | TATTTGATTTGCGCCTGCAC |
| qR *ycgF* | CAATGGCTTCAAAAGCGATT |
| qF *soxS* | TGTCCCATCAGAAAATTATTCAGG |
| qR *soxS* | TGGGAGTGCGATCAAACTGC |
| qF *yidB* | ATGCGGGGAAATATCAGGCT |
| qR *yidB* | TGGCTTGTGGTGACACTTCA |
| qF *dnaK* | GACCGACGTTAACCTGCCATA |
| qR *dnaK* | TGAACAGCAGCACCGATTGC |
| qF *spoT* | CGTGATCGTCAATGATTCTGACAC |
| qR *spoT* | TCCAGCAGGCTTTGCATCCA |
| qF ybgK | GGTCGCGGGTGGTATTGATG |
| qR ybgK | TGCCCCTGTAAGCGATAGCC |
| qF *rpoD* | GATCAACGACATGGGCATTCAGGTG |
| qR *rpoD* | CTTCTTCCAGCGTGTAGTCGGTGTTCATA |
| qF *yeaP* | GCTTTTTCAGCGATGAAGTCC |
| qR *yeaP* | GATTCGGTAGCCCGGTTAAC |
| qF *uvrA* | GCTATCTCCGACATGAGCATTG |
| qR *uvrA* | ACCCAACAGGCGCTCGTTAT |
| qF *mgtA* | AAACAGAAAGTGATCGTCAAACATC |
| qR *mgtA* | ACGCTCGAAATCAAACGGAA |
| qF *lpxC* | TGTCTGGTCAACGAGCATGAT |
| qR *lpxC* | CGGATGGTTAAAATCGATGG |
| qF *mlrA* | GACTTACCTGCAAAGCGGCA |
| qR *mlrA* | CCTTGTTGACTGGCAATCCA |
