# Supplementary material for: DNA, Cell Wall and General Oxidative Damage Underlie the Tellurite/Cefotaxime Synergistic Effect in Escherichia coli
Source: PLoS One. 2013 Nov 18;8(11):e79499. doi: 10.1371/journal.pone.0079499 (PMC3832599; doi:10.1371/journal.pone.0079499)
Supplement: Table S2 — (DOCX) [file pone.0079499.s006.docx]

**Table S2.** Genes differentially expressed in *E. coli* exposed to tellurite (A), CTX (B) and tellurite/CTX (C). Global transcriptional changes were determined as described in Methods.

**A**

Tellurite, induced

| Symbol | Description | Fold change | |
| --- | --- | --- | --- |
| *soxS* | DNA-binding transcriptional dual regulator | | 4.7 |
| *yeaP* | predicted diguanylate cyclase | | 3.7 |
| *ybgK* | predicted enzyme subunit | | 3.6 |
| *dnaK* | molecular chaperone DnaK | | 2.8 |
| *marR* | DNA-binding transcriptional repressor of multiple antibiotic resistance | | 2.8 |
| *yhiI* | "Hypothetical protein yhiI precursor" | | 2.6 |
| *yagL* | CP4-6 prophage; DNA-binding protein | | 2.5 |
| *gmk* | guanylate kinase | | 2.5 |
| *yqgA* | predicted inner membrane protein | | 2.5 |
| *shiA* | shikimate transporter | | 2.5 |
| *ybfH* | hypothetical protein | | 2.5 |
| *rfaB* | UDP-D-galactose:(glucosyl)lipopolysaccharide-1, 6-D-galactosyltransferase | | 2.5 |
| *rnr* | exoribonuclease R, RNase R | | 2.4 |
| *fumA* | fumarate hydratase (fumarase A), aerobic Class I | | 2.3 |
| *yahD* | predicted transcriptional regulator with ankyrin domain | | 2.2 |
| *lnt* | apolipoprotein N-acyltransferase | | 2.2 |
| *miaA* | tRNA delta(2)-isopentenylpyrophosphate transferase | | 2.2 |
| *sfaC* | "putative F1C and S fimbrial switch regulatory protein" | | 2.1 |
| *yfhQ* | predicted methyltransferase | | 2.1 |
|  |  | |  |

Tellurite, repressed

| Symbol | Description | | Fold change |
| --- | --- | --- | --- |
| *yadT* | vitamin B12-transporter protein BtuF | | -3.5 |
| *cspA* | major cold shock protein | | -2.7 |
| *cspG* | DNA-binding transcriptional regulator | | -2.5 |
| *narG* | "nitrate reductase 1, alpha subunit " | | -2.5 |
| *yfaQ* | hypothetical protein | | -2.4 |
| *alkB* | oxidative demethylase of N1-methyladenine or N3-methylcytosine DNA lesions | | -2.4 |
| *tehA* | potassium-tellurite ethidium and proflavin transporter | | -2.4 |
| *yddE* | "Hypothetical protein yddE (ORFB)" | | -2.3 |
| *yrdA* | "Protein yrdA" | | -2.3 |
| *manZ* | mannose-specific enzyme IID component of PTS | | -2.3 |
| *purR* | DNA-binding transcriptional repressor, hypoxanthine-binding | | -2.2 |
| *yjdA* | conserved protein with nucleoside triphosphate hydrolase domain | | -2.2 |
| *rseB* | periplasmic negative regulator of sigmaE | | -2.2 |
| *sdaC* | predicted serine transporter | | -2.2 |
| *sgaE* | "Probable sugar isomerase sgaE" | | -2.2 |
| *glpB* | anaerobic glycerol-3-phosphate dehydrogenase subunit B | | -2.1 |
| *grxB* | glutaredoxin 2 (Grx2) | | -2.1 |
| *argC* | "N-acetyl-gamma-glutamyl-phosphate reductase" | | -2.1 |
| *insA-7* | KpLE2 phage-like element; IS1 repressor protein InsA | | -2.1 |
| *ptsG* | fused glucose-specific PTS enzymes: IIB | | -2.1 |
| *dcuS* | sensory histidine kinase in two-component regulatory system with DcuR | | -2.0 |
| *hsdR* | endonuclease R | | -2.0 |
| *yghW* | hypothetical protein | | -2.0 |
| *fabB* | 3-oxoacyl-(acyl carrier protein) synthase | | -2.0 |
|  |  |  | |

**B**

CTX, induced

| Symbol | Description | Fold change |
| --- | --- | --- |
| *spoT* | bifunctional (p)ppGpp synthetase II/ guanosine-3',5'-bis pyrophosphate 3'-pyrophosphohydrolase | 4.5 |
| *ycgF* | "Hypothetical protein ycgF" | 3.9 |
| *yhdA* | conserved inner membrane protein | 3.8 |
| *uvrA* | excinuclease ABC subunit A | 3.7 |
| *ureE* | putative urease accessory protein E \| putative urease accessory protein E | 3.0 |
| *yaaH* | conserved inner membrane protein associated with acetate transport | 2.8 |
| *ydcD* | hypothetical protein | 2.6 |
| *polA* | DNA polymerase I | 2.6 |
| *ycfL* | hypothetical protein | 2.6 |
| *chuY* | "orf; hypothetical protein" | 2.5 |
| *ydgK* | conserved inner membrane protein | 2.4 |
| *thiH* | thiamine biosynthesis protein ThiH | 2.4 |
| *yjgP* | conserved inner membrane protein | 2.4 |
| *ygjM* | predicted DNA-binding transcriptional regulator | 2.4 |
| *purA* | adenylosuccinate synthetase | 2.4 |
| *ydcJ* | hypothetical protein | 2.3 |
| *fdoI* | formate dehydrogenase-O, cytochrome b556 subunit | 2.2 |
| *yeaB* | predicted NUDIX hydrolase | 2.2 |
| *ybgK* | predicted enzyme subunit | 2.2 |
| *yabF* | "Putative NAD(P)H oxidoreductase yabF" | 2.2 |
| *ycfK* | e14 prophage; predicted protein | 2.2 |
| *yjiN* | "Hypothetical protein yjiN" | 2.1 |
| *gspC* | general secretory pathway component, cryptic | 2.1 |
| *smpA* | small membrane lipoprotein | 2.1 |
| *ygeD* | predicted inner membrane protein | 2.1 |
| *yfiR* | hypothetical protein | 2.1 |
| *intZ* | CPZ-55 prophage; predicted integrase | 2.0 |
| *guaC* | guanosine 5'-monophosphate oxidoreductase | 2.0 |
| *nikC* | nickel transporter subunit | 2.0 |
| *secG* | protein-export membrane protein | 2.0 |
| *yfeX* | "Hypothetical protein yfeX" | 2.0 |
| *ynfM* | predicted transporter | 2.0 |

CTX, repressed

| Symbol | | Description | Fold change |  | Symbol | Description | Fold change |
| --- | --- | --- | --- | --- | --- | --- | --- |
| *ldcA* | L,D-carboxypeptidase A | | -3.9 |  | *ybjP* | predicted lipoprotein | -2.3 |
| *arsC* | arsenate reductase | | -3.9 |  | *ybgJ* | predicted enzyme subunit | -2.3 |
| *yehC* | "putative chaperone protein " | | -3.4 |  | *pspE* | thiosulfate:cyanide sulfurtransferase rhodanese) | -2.3 |
| *purF* | Amidophosphoribosyltransferase | | -3.4 |  | *rpoN* | DNA-directed RNA polymerase subunit N | -2.3 |
| *dmsB* | dimethyl sulfoxide reductase, anaerobic, subunit B | | -3.3 |  | *ydjC* | hypothetical protein | -2.3 |
| *yfbH* | hypothetical protein | | -3.2 |  | *manY* | mannose-specific enzyme IIC component of PTS | -2.3 |
| *nrdF* | "Ribonucleoside-diphosphate reductase 2 beta chain" | | -3.2 |  | *ykgB* | conserved inner membrane protein | -2.2 |
| *yieL* | predicted xylanase | | -3.1 |  | *yehW* | predicted transporter subunit: membrane component of ABC superfamily | -2.2 |
| *nupG* | "transport of nucleosides, permease protein " | | -3.0 |  | *hscA* | "Chaperone protein hscA (Hsc66)" | -2.2 |
| *wbdP* | "glycosyl transferase " | | -2.9 |  | *garD* | (D)-galactarate dehydrogenase | -2.2 |
| *afuB* | "putative permease component of transport system for ferric iron " | | -2.9 |  | *yheR* | glutathione-regulated potassium-efflux system ancillary protein | -2.2 |
| *phnH* | carbon-phosphorus lyase complex subunit | | -2.8 |  | *trpA* | tryptophan synthase subunit alpha | -2.2 |
| *ykfC* | CP4-6 prophage; conserved protein | | -2.8 |  | *yagX* | predicted aromatic compound dioxygenase | -2.2 |
| *aldA* | aldehyde dehydrogenase A, NAD-linked | | -2.7 |  | *pflC* | pyruvate formate lyase II activase | -2.2 |
| *yecN* | predicted inner membrane protein | | -2.6 |  | *htrE* | predicted outer membrane usher protein | -2.2 |
| *ycgG* | conserved inner membrane protein | | -2.6 |  | *adiY* | DNA-binding transcriptional activator | -2.2 |
| *yibH* | hypothetical protein | | -2.6 |  | *ybjI* | hypothetical protein | -2.2 |
| *nfo* | endonuclease IV | | -2.6 |  | *livF* | leucine/isoleucine/valine transporter subunit | -2.2 |
| *yffB* | hypothetical protein | | -2.5 |  | *yecP* | "Hypothetical protein yecP" | -2.2 |
| *puuP* | putrescine importer | | -2.5 |  | *ybhS* | predicted transporter subunit: membrane component of ABC superfamily | -2.2 |
| *ygcL* | hypothetical protein | | -2.5 |  | *bcsC* | cellulose synthase subunit | -2.2 |
| *yfaW* | predicted enolase | | -2.5 |  | *glyA* | serine hydroxymethyltransferase | -2.1 |
| *iap* | aminopeptidase in alkaline phosphatase isozyme conversion | | -2.5 |  | *wcaL* | predicted glycosyl transferase | -2.1 |
| *ycbF* | predicted periplasmic pilini chaperone | | -2.5 |  | *ydcF* | "Protein ydcF" | -2.1 |
| *yccT* | hypothetical protein | | -2.5 |  | *gutM* | DNA-binding transcriptional activator of glucitol operon | -2.1 |
| *glvB* | arbutin specific enzyme IIB component of PTS | | -2.5 |  | *phoP* | DNA-binding response regulator in two-component regulatory system with PhoQ | -2.1 |
| *hemX* | predicted uroporphyrinogen III methylase | | -2.5 |  | *yadL* | "putative fimbrial protein " | -2.1 |
| *yegV* | predicted kinase | | -2.5 |  | *ygiS* | predicted transporter subunit: periplasmic-binding component of ABC superfamily | -2.1 |
| *yjgX* | KpLE2 phage-like element; predicted protein | | -2.5 |  | *yfcA* | conserved inner membrane protein | -2.1 |
| *idnD* | L-idonate 5-dehydrogenase, NAD-binding | | -2.4 |  | *cysQ* | "CysQ protein" | -2.1 |
| *ybiV* | predicted hydrolase | | -2.4 |  | *xylR* | DNA-binding transcriptional activator, xylose-binding | -2.1 |
| *ybhE* | 6-phosphogluconolactonase | | -2.4 |  | *mutT* | nucleoside triphosphate pyrophosphohydrolase, marked preference for dGTP | -2.1 |
| *ycfT* | "Hypothetical protein ycfT" | | -2.4 |  | *ytfE* | "Hypothetical protein ytfE" | -2.1 |
| *yjeJ* | hypothetical protein | | -2.4 |  | *napD* | assembly protein for periplasmic nitrate reductase | -2.1 |
| *ypeA* | "Hypothetical protein ypeA" | | -2.4 |  | *ydfC* | Qin prophage; predicted protein | -2.1 |
| *allC* | N-carbamoyl-L-amino acid amidohydrolase | | -2.4 |  | *ybbS* | DNA-binding transcriptional activator of the allD operon | -2.1 |
| *yejM* | predicted hydrolase, inner membrane | | -2.4 |  | *yjgG_2* | "hypothetical protein " | -2.0 |
| *yojL* | predicted thiamine biosynthesis lipoprotein | | -2.3 |  | *tufB* | protein chain elongation factor EF-Tu (duplicate of tufA) | -2.0 |
| *ygjE* | predicted tartrate:succinate antiporter | | -2.3 |  | *glgC* | glucose-1-phosphate adenylyltransferase | -2.0 |
| *yfhM* | hypothetical protein | | -2.3 |  | *rseC* | RseC protein involved in reduction of the SoxR iron-sulfur cluster | -2.0 |
| *yjiD* | "Hypothetical protein yjiD" | | -2.3 |  | *escR* | "escR " | -2.0 |
| *yrdB* | hypothetical protein | | -2.3 |  | *ybbP* | predicted inner membrane protein | -2.0 |

**C**

Tellurite/CTX, induced

| Symbol | Description | Fold change |
| --- | --- | --- |
| *soxS* | DNA-binding transcriptional dual regulator | 7.8 |
| *marR* | DNA-binding transcriptional repressor of multiple antibiotic resistance | 3.5 |
| *dnaK* | molecular chaperone DnaK | 3.0 |
| *ybgK* | predicted enzyme subunit | 3.0 |
| *ycjO* | predicted sugar transporter subunit: membrane component of ABC superfamily | 2.9 |
| *mgtA* | magnesium transporter | 2.8 |
| *yffI* | predicted carboxysome structural protein with predicted role in ethanolamine utilization | 2.7 |
| *yfhQ* | predicted methyltransferase | 2.6 |
| *rpoD* | RNA polymerase sigma factor | 2.6 |
| *htrL* | hypothetical protein | 2.5 |
| *miaA* | tRNA delta(2)-isopentenylpyrophosphate transferase | 2.5 |
| *clpB* | protein disaggregation chaperone | 2.5 |
| *dos* | cAMP phosphodiesterase, heme-regulated | 2.4 |
| *glcC* | DNA-binding transcriptional dual regulator, glycolate-binding | 2.3 |
| *ybhG* | hypothetical protein | 2.3 |
| *glnH* | glutamine ABC transporter periplasmic protein | 2.2 |
| *ygaC* | hypothetical protein | 2.2 |
| *ygfO* | predicted transporter | 2.2 |
| *gmr* | modulator of Rnase II stability | 2.2 |
| *acrE* | cytoplasmic membrane lipoprotein | 2.2 |
| *dgoA* | 2-dehydro-3-deoxy-6-phosphogalactonate aldolase | 2.2 |
| *yidH* | conserved inner membrane protein | 2.1 |
| *hycD* | hydrogenase 3, membrane subunit | 2.1 |
| *yoaE* | fused predicted membrane protein/conserved protein | 2.1 |
| *yaeL* | zinc metallopeptidase | 2.1 |
| *marB* | hypothetical protein | 2.0 |
| *iscU* | scaffold protein | 2.0 |
| *ybgC* | predicted acyl-CoA thioesterase | 2.0 |
| *gmk* | guanylate kinase | 2.0 |

Tellurite/CTX, repressed

| Symbol | Description | Fold change |
| --- | --- | --- |
| *yhbP* | hypothetical protein | -5.2 |
| *cspB* | Qin prophage; cold shock protein | -5.0 |
| *yjgL* | hypothetical protein | -4.3 |
| *cpdA* | cyclic 3',5'-adenosine monophosphate phosphodiesterase | -3.9 |
| *yiaW* | "hypothetical protein " | -3.7 |
| *cspA* | major cold shock protein | -3.2 |
| *yibO* | phosphoglyceromutase | -3.1 |
| *ycaM* | predicted transporter | -2.9 |
| *yciW* | predicted oxidoreductase | -2.9 |
| *racC* | Rac prophage; predicted protein | -2.9 |
| *ydgI* | predicted arginine/ornithine antiporter transporter | -2.8 |
| *emrA* | multidrug efflux system | -2.7 |
| *paaG* | enoyl-CoA hydratase | -2.6 |
| *tdcD* | "acetate/propionate kinase " | -2.6 |
| *ycjQ* | "putative oxidoreductase " | -2.5 |
| *uvrY* | response regulator | -2.4 |
| *gdhA* | glutamate dehydrogenase | -2.4 |
| *yjdB* | "Hypothetical protein yjdB" | -2.4 |
| *cspG* | DNA-binding transcriptional regulator | -2.3 |
| *chbR* | DNA-binding transcriptional dual regulator | -2.3 |
| *ppnK* | inorganic polyphosphate/ATP-NAD kinase | -2.3 |
| *dhaH* | fused predicted dihydroxyacetone-specific PTS | -2.3 |
| *ygfT* | fused predicted oxidoreductase: Fe-S | -2.2 |
| *nikD* | "ATP-binding protein of nickel transport system " | -2.2 |
| *yibF* | predicted glutathione S-transferase | -2.2 |
| *yfdG* | CPS-53 (KpLE1) prophage; bactoprenol-linked glucose translocase (flippase) | -2.1 |
| *nanT* | "transport; Murein sacculus, peptidoglycan" | -2.1 |
| *dgoD* | galactonate dehydratase | -2.1 |
| *pykF* | "enzyme; Energy metabolism, carbon: Glycolysis" | -2.1 |
| *ybcM* | DLP12 prophage; predicted DNA-binding transcriptional regulator | -2.1 |
| *nrdB* | ribonucleotide-diphosphate reductase beta subunit | -2.1 |
| *ydiN* | predicted transporter | -2.0 |
| *yfaL* | adhesin | -2.0 |
