# Supplementary material for: DNA, Cell Wall and General Oxidative Damage Underlie the Tellurite/Cefotaxime Synergistic Effect in Escherichia coli
Source: PLoS One. 2013 Nov 18;8(11):e79499. doi: 10.1371/journal.pone.0079499 (PMC3832599; doi:10.1371/journal.pone.0079499)
Supplement: Table S3 — (DOCX) [file pone.0079499.s007.docx]

**Table S3**. Assessment of transcriptional changes mediated by two different tellurite concentrations. *E. coli* cells were exposed to tellurite 0.05 or 0.5 µg ml^-1^ and expression changes were determined by DNA microarrays.

| Gene | Log-fold change  (tellurite 0.05 µg ml^-1^) | Log-fold change  (tellurite 0.5 µg ml^-1^) |
| --- | --- | --- |
| *soxS* | 2.24 | 3.24 |
| *marR* | 1.49 | 2.24 |
| *gmK* | 1.32 | 2.10 |
| *cspA* | -1.43 | -2.24 |
