# Supplementary material for: DNA, Cell Wall and General Oxidative Damage Underlie the Tellurite/Cefotaxime Synergistic Effect in Escherichia coli
Source: PLoS One. 2013 Nov 18;8(11):e79499. doi: 10.1371/journal.pone.0079499 (PMC3832599; doi:10.1371/journal.pone.0079499)
Supplement: Table S4 — (DOCX) [file pone.0079499.s008.docx]

Table S4. Genes differentially expressed in *E. coli* exposed to tellurite (T), CTX (C) or tellurite/CTX (T/C).

| Expression | | Treatment |  | Gene | Function |
| --- | --- | --- | --- | --- | --- |
| I | T |  | T/C | *soxS* | DNA-binding transcriptional dual regulator |
| I | T |  |  | *yeaP* | predicted diguanylate cyclase |
| I | T | C | T/C | *ybgK* | predicted enzyme subunit |
| I | T |  | T/C | *dnaK* | molecular chaperone DnaK |
| I | T |  | T/C | *marR* | DNA-binding transcriptional repressor of multiple antibiotic resistance |
| I | T |  |  | *yhiI* | Hypothetical protein yhiI precursor" |
| I | T |  |  | *yagL* | CP4-6 prophage; DNA-binding protein |
| I | T |  | T/C | *gmk* | guanylate kinase |
| I | T |  |  | *yqgA* | predicted inner membrane protein |
| I | T |  |  | *shiA* | shikimate transporter |
| I | T |  |  | *ybfH* | hypothetical protein |
| I | T |  |  | *rfaB* | UDP-D-galactose:(glucosyl)lipopolysaccharide-1, 6-D-galactosyltransferase |
| I | T |  |  | *Rnr* | exoribonuclease R, RNase R |
| I | T |  |  | *fumA* | fumarate hydratase (fumarase A), aerobic Class I |
| I | T |  |  | *yahD* | predicted transcriptional regulator with ankyrin domain |
| I | T |  |  | *Lnt* | apolipoprotein N-acyltransferase |
| I | T |  | T/C | *miaA* | tRNA delta(2)-isopentenylpyrophosphate transferase |
| I | T |  |  | *sfaC* | putative F1C and S fimbrial switch regulatory protein |
| I | T |  | T/C | *yfhQ* | predicted methyltransferase |
| I |  | C |  | *spoT* | guanosine-3',5'-bis pyrophosphate 3'-pyrophosphohydrolase |
| I |  | C |  | *ycgF* | Hypothetical protein ycgF |
| I |  | C |  | *yhdA* | conserved inner membrane protein |
| I |  | C |  | *uvrA* | excinuclease ABC subunit A |
| I |  | C |  | *ureE* | putative urease accessory protein E \| putative urease accessory protein E |
| I |  | C |  | *yaaH* | conserved inner membrane protein associated with acetate transport |
| I |  | C |  | *ydcD* | hypothetical protein |
| I |  | C |  | *polA* | DNA polymerase I |
| I |  | C |  | *ycfL* | hypothetical protein |
| I |  | C |  | *chuY* | "orf; hypothetical protein" |
| I |  | C |  | *ydgK* | conserved inner membrane protein |
| I |  | C |  | *thiH* | thiamine biosynthesis protein ThiH |
| I |  | C |  | *yjgP* | conserved inner membrane protein |
| I |  | C |  | *ygjM* | predicted DNA-binding transcriptional regulator |
| I |  | C |  | *purA* | adenylosuccinate synthetase |
| I |  | C |  | *ydcJ* | hypothetical protein |
| I |  | C |  | *fdoI* | formate dehydrogenase-O, cytochrome b556 subunit |
| I |  | C |  | *yeaB* | predicted NUDIX hydrolase |
| I |  | C |  | *yabF* | Putative NAD(P)H oxidoreductase yabF |
| I |  | C |  | *ycfK* | e14 prophage; predicted protein |
| I |  | C |  | *yjiN* | Hypothetical protein yjiN |
| I |  | C |  | *gspC* | general secretory pathway component, cryptic |
| I |  | C |  | *smpA* | small membrane lipoprotein |
| I |  | C |  | *ygeD* | predicted inner membrane protein |

Table S4 (cont.)

| I |  | C |  | *yfiR* | hypothetical protein |
| --- | --- | --- | --- | --- | --- |
| I |  | C |  | *intZ* | CPZ-55 prophage; predicted integrase |
| I |  | C |  | *guaC* | guanosine 5'-monophosphate oxidoreductase |
| I |  | C |  | *nikC* | nickel transporter subunit |
| I |  | C |  | *secG* | protein-export membrane protein |
| I |  | C |  | *yfeX* | Hypothetical protein yfeX |
| I |  | C |  | *ynfM* | predicted transporter |
| I |  |  | T/C | *ycjO* | predicted sugar transporter subunit: membrane component of ABC superfamily |
| I |  |  | T/C | *mgtA* | magnesium transporter |
| I |  |  | T/C | *yffI* | predicted carboxysome structural protein with predicted role in ethanolamine utilization |
| I |  |  | T/C | *rpoD* | RNA polymerase sigma factor |
| I |  |  | T/C | *htrL* | hypothetical protein |
| I |  |  | T/C | *clpB* | protein disaggregation chaperone |
| I |  |  | T/C | *Dos* | cAMP phosphodiesterase, heme-regulated |
| I |  |  | T/C | *glcC* | DNA-binding transcriptional dual regulator, glycolate-binding |
| I |  |  | T/C | *ybhG* | hypothetical protein |
| I |  |  | T/C | *glnH* | glutamine ABC transporter periplasmic protein |
| I |  |  | T/C | *ygaC* | hypothetical protein |
| I |  |  | T/C | *ygfO* | predicted transporter |
| I |  |  | T/C | *gmr* | modulator of Rnase II stability |
| I |  |  | T/C | *acrE* | cytoplasmic membrane lipoprotein |
| I |  |  | T/C | *dgoA* | 2-dehydro-3-deoxy-6-phosphogalactonate aldolase |
| I |  |  | T/C | *yidH* | conserved inner membrane protein |
| I |  |  | T/C | *hycD* | hydrogenase 3, membrane subunit |
| I |  |  | T/C | *yoaE* | fused predicted membrane protein/conserved protein |
| I |  |  | T/C | *yaeL* | zinc metallopeptidase |
| I |  |  | T/C | *marB* | hypothetical protein |
| I |  |  | T/C | *iscU* | scaffold protein |
| I |  |  | T/C | *ybgC* | predicted acyl-CoA thioesterase |
| R | T |  | T/C | *cspG* | DNA-binding transcriptional regulator |
| R | T |  | T/C | *cspA* | major cold shock protein |

I, induced; R, repressed
